# Household plastic waste habits and attitudes: A pilot study in the city of Valencia

**DOI:** 10.1177/0734242X21996415

**Published:** 2021-03-21

**Authors:** Isabelle Roche Cerasi, Francisco V Sánchez, Iris Gallardo, Miguel Á Górriz, Paula Torrijos, César Aliaga, Jerónimo Franco

**Affiliations:** 1 Department of Mobility and Economics, SINTEF Community, Norway; 2 ITENE, Instituto Tecnológico del Embalaje, Transporte y Logística, Spain; 3 SAV, Agricultores de la Vega de Valencia, Spain

**Keywords:** Plastic waste, waste characterization, circular economy, public attitude, smart technologies

## Abstract

Bearing in mind that only 42% of plastic packaging post-consumer waste is recycled in Europe, the European Directive 2018/852 established the key target of a 55% plastic packaging waste recycling rate by 2030. For this reason, PlastiCircle, funded by the European Union’s Horizon 2020 research and innovation program project, aims to foster the recycling of packaging, improve all stages of the waste collection, and promote responsible consumption. Three European cities have been selected as locations for pilot implementation: Valencia (Spain), Utrecht (The Netherlands) and Alba Iulia (Romania). The main objective of the present study has been to evaluate the participants’ opinion and attitudes on plastic recycling. This paper presents the results from the district of San Marcelino in the city of Valencia, the first PlastiCircle pilot to face the challenges of encouraging households to participate more in plastic waste sorting and recycling.

## Introduction

Plastic production has increased over the last century, currently reaching a production of 61.8 million tonnes in Europe, becoming the preferred material of the industry. The production of oil products, which are flexible and relatively cheap, generate massive production that ends up flooding the planet with plastic (PlasticsEurope, 2019). More than 29.1 million tonnes of plastic post-consumer waste were collected in Europe, with only 32.5% being recycled, whereas 42.6% was incinerated and 24.9% landfilled (Eurostat, 2018).

Packaging is the main plastic waste fraction, with 15.8 million tonnes generated in the European Union per year (Eurostat, 2018). Additionally, the recycling rate of domestic packages is limited by waste quality and the citizens’ behaviour and environmental awareness. Moreover, the high variety of polymers in plastic collected causes life cycle issues. Only 42% of packaging waste is recycled in Europe (PlasticsEurope, 2019). This situation is still far from the objective of 55% by 2030 established in the European Plastic Strategy and the European Directive 2018/852 (EUR-Lex, 2018).

**Figure 1.**
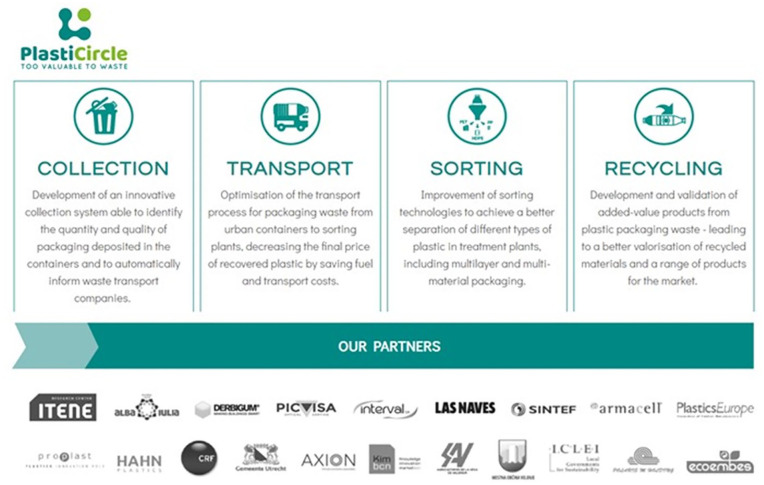
The PlastiCircle approach.

In order to achieve this target, the European Union’s Horizon 2020 research and innovation program project PlastiCircle (2017–2021) aimed to transform waste into valuable products: better and cheaper secondary raw materials, improved domestic packaging waste collection and treatment, increased recycling rate, as well as better recovery and valorisation. Figure 1 presents the PlastiCircle approach with the involved partners.

Twenty European partners have joined forces in developing smart containers to increase collection rates of plastic waste, cost-effective waste transport systems connected to internet of things cloud platforms, innovative optical sorting technologies, and new value-added recycled plastic products. ITENE, the packaging, transport, and logistics research centre in Valencia, was the coordinator of the project and responsible for the development of the label dispenser. LAS NAVES, the non-profit foundation promoting social and urban innovations, was responsible for the information stands and for managing the gift exchange process. SAV, the Spanish waste management company, was responsible for the characterization of the trash bag content. SINTEF, the Norwegian research institute, was responsible for the evaluation of the pilot studies.

PlastiCircle demonstrated the concept in three countries: Valencia (Spain), Utrecht (The Netherlands) and Alba Iulia (Romania). The objective was to support the ambitious target for recycling 75% of packaging waste by 2030 of the European Commission in the Circular Economy Package (European Commission, 2019b). The circular economy is a model which implies an increase in recycling rates for effective and environmentally waste management systems (Ragossnig and Schneider, 2019). However, this requires reducing the contamination level of waste plastics (Horodytska et al., 2018) hampering effective identification, segregation, and purification of various types of plastics (Siddique et al., 2008, 2010). Communication strategies are crucial to raise public awareness about their responsibility and what to recycle and reuse (Stahel, 2016).

**Figure 2.**
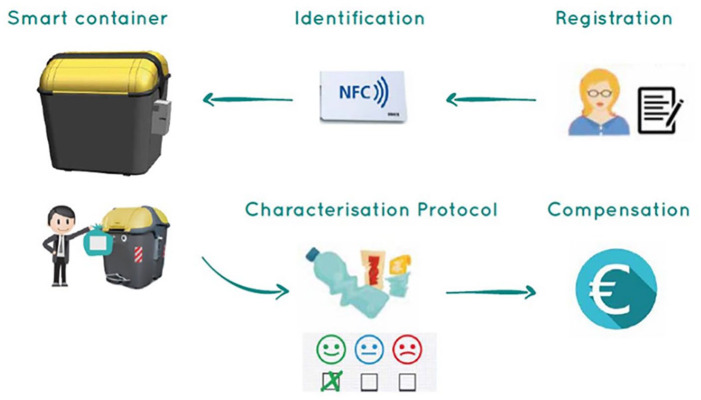
Registration to compensation process.

The objective of the present study was to evaluate the participants’ opinion on the pilot study carried out in Valencia and to examine their attitudes towards recycling behaviour. Figure 2 shows the entire process planned in PlastiCircle from the registration of participants to the compensation procedure. The participants, registered thanks to the use of near field communication (NFC) cards, had to fill trash bags with recyclables, to stick a label on them and to put them in the container. They received compensation after the characterization of their bags.

Previous studies have shown that the theory of planned behaviour (TPB) provides a theoretical framework for identifying determinants of recycling behaviour (Kumar, 2017; Botetzagias et al., 2015; Cheung et al., 1999; Greaves et al., 2013; Khan et al., 2019; Mahmud and Osman, 2010; Nigbur et al., 2010; Oztekin et al., 2017; Park and Ha, 2004; Tonglet et al., 2004; Yin et al., 2014). The TPB theory showed that attitude, subjective norms, and perceived behavioural control are decisive for predicting the individuals’ behavioural intention (Ajzen, 1991).

Khan et al. (2019), Botetzagias et al. (2015) and Mahmud and Osman (2010) conducted studies based on TPB and confirmed that awareness consequences and convenience are major predictors of return or recycling intention. The review conducted by Heidbreder et al. (2019) suggested that although individuals are aware of the negative effects of plastic consumption on health and the environment, their consumer habits, advantages of plastic use, and situational factors often slowed down their willingness to reduce their plastic consumption. Tonglet et al. (2004) and Cheung et al. (1999) showed that pro-recycling attitudes are influenced by access to facilities and environmental knowledge. Nigbur et al. (2010) and Greaves et al. (2013) demonstrated the importance of involving the population in new recycling processes to ensure relevant behaviours and to make the citizens identify themselves as recyclers.

Sociodemographic variables predicting plastic practices are gender, income, and education, with women being more willing to shift to eco-friendly alternatives than men (Jeżewska-Zychowicz and Jeznach, 2015; Sharp et al., 2010). However, other studies did not find significant differences between age and gender groups (Botetzagias et al., 2015; Greaves et al., 2013). Afroz et al. (2017) also found that in Malaysia, older people, higher educated and high-income groups are more willing to participate in no plastic bag campaign than their counterparts. However, Elgaaïed-Gambier (2016) argued, based on a study on consumers’ perception of over-packaged products, that younger French consumers could be more willing to give up their own convenience to preserve the environment.

The pay-as-you-throw (PAYT) principle implies that consumers who throw away more, should pay more. This principle was successful in many European countries and beyond, for example, to reduce the amount of single-use plastic bags (Botetzagias et al., 2020; Chamizo-González et al., 2018; Elia et al., 2015; Wagner, 2017). The introduction of the PAYT charge operates as a stimulus for waste reduction and an incentive for illegal dumping (Botetzagias et al., 2020). The European Environment Agency (EEA) (2019) also stated that although the single-use plastic bag charge provided impressive results, countries should be encouraged to diversify their implemented measures (EEA, 2019).

Another successful incentive such as refusing to pick up contaminated recycle bins has shown good results (WasteZero.com, 2020). Bans and increased costs as well as awareness interventions seem to be adapted measures to reduce plastic consumption and increase return/recycling behaviour.

Education level, age and household income were found to be significant factors for willingness to pay for recycled and recycling products (Siddique et al., 2010; Vasileva and Ivanova, 2014).

## Materials and method

### Participants

The pilot was limited to the San Marcelino district in the city of Valencia, a small geographic district of around 10,000 inhabitants or 5000 households. Diverse communication actions were launched for informing the citizens about the pilot and to increase awareness about the importance of circular economy and recycling. Leaflets and posters were distributed, and trained staff were present at the district two days a week during the entire pilot. The total number of registered families was 556, and 1464 citizens were involved in the different activities of the pilot. The proportion of females and males was respectively 53.1% and 46.9%. The participants were registered through a form on the website developed by LAS NAVES and SAV (Supermarcelina.com). Each participant received a personal identification 4-digit number and could check on the platform their performance and their compensation ecopoints.

### Monitoring period

The pilot in Valencia ran officially from May to October 2019. The registration started before the calendar week 20 with the installation of dispensers on the side of containers and antennas in the neighbourhood.

### Questionnaires

The participants were asked to answer a questionnaire before and after the pilot study. The pre-questionnaire focused on their opinion on the current domestic waste management and plastic collection in general, their waste sorting habits, their perceived benefits of recycling for the society, their willingness to pay for recycling and their concern about waste data privacy issues.

The post-questionnaire was divided into several parts to evaluate the pilot; these parts covered their opinion about the easiness of sorting the recyclable packages at home, using the orange trash bags and the label dispenser. The participants were also asked to provide their opinion about the compensation system and whether the PAYT principle would be a measure they could accept. They were also asked to evaluate the PlastiCircle training sessions and communication. The final parts focused on their opinion about the project in general, and what could have been done better.

### Sample

Descriptive statistics were used to reveal the characteristics of the sample. The results were analysed with the IBM SPSS statistics 25 software. By convention, the cut-off point for the statistical results is a *p*-value of 0.05. The participants were asked to fill out a questionnaire before and after the pilot; 116 and 114 participants respectively answered the pre- and post-questionnaire, and 54 of them answered both the pre- and post-questionnaires. For a 95% confidence level, the total number of respondents for the questionnaires to be reached with a ±10% margin of error was calculated to be over around 100 respondents (Brace et al., 2016). The survey response rates of approximately 21% were therefore considered to be high enough to minimize sampling bias.

Table 1 illustrates the proportion of males and females per age group. The pilot groups are composed of individuals aged from 18 to 74 years. The results showed that slightly less than half of the respondents are middle-aged adults (35–54 years old) in both groups (respectively 49.1% and 45.6%). The proportions of females in the pre- and post-pilot groups are respectively 61.2% and 63.2%, whereas, for the males, they are 28.4% and 28.9%; 10.3% and 7.9% of the respondents preferred not to answer the question about the gender in both groups. Differences between men and women should be interpreted with caution in this study, because the number of women in the groups is high and might affect the reliability of the comparison between genders. Independent tests showed that there are no significant gender, age, and education differences between the pre- and post-pilot groups (p > .05).

**Table 1.** Distribution of respondents in the sample per gender and age groups.

Age group (years)	Gender											
	Female				Male				No answer			
Sample	Pre		Post		Pre		Post		Pre		Post	
	*n*	%	*n*	%	*n*	%	*n*	%	*n*	%	*n*	%
18–24	2	2.8	2	2.8	1	3.0	1	3.0	0	0.0	0	0.0
25–34	16	22.5	12	16.7	6	18.2	2	6.1	0	0.0	0	0.0
35–44	23	32.4	17	23.6	6	18.2	6	18.2	1	8.3	0	0.0
45–54	19	26.8	23	31.9	7	21.2	4	12.1	1	8.3	2	22.2
55–64	7	9.9	13	18.1	7	21.2	13	39.4	1	8.3	0	0
65–74	4	5.6	3	4.2	6	18.2	7	21.2	0	0.0	0	0.0
75 and more	0	0.0	0	0.0	0	0.0	0	0.0	0	0.0	0	0.0
No answer	0	0.0	2	2.8	0	0.0	0	0.0	9	75.0	7	77.8
Total	71	100	72	100	33	100	33	100	12	100	9	100

For the education levels (basic level = no studies, primary and secondary school, professional formation; high level = university or doctorate), 54.3% and 58.8% had a basic education level in both groups, respectively. Slightly over half of the respondents (50.9% and 52.6%) were employed either full time or part time. According to the municipality, the population living in the San Marcelino district is mostly composed of working-class households with basic education levels.

## Research findings

### Domestic waste management

The results indicated that a large share of the pre-sample was in general satisfied with the current domestic waste management in their municipality (68.1%). Of the 116 respondents, 61.2% also strongly agreed or agreed that they are well informed about the domestic management system in their municipality.

Valencia is collecting and separating eight different domestic waste streams:

Paper and cardboard
Light packaging: plastic, cans, and beverage cartons
Glass
Used oil
Waste electrical and electronic equipment
Bulky products
Other waste
Organic (started as a trial pilot).

The municipal containers are of different colours according to waste type: municipal solid waste (grey), light or plastic packaging (yellow), glass (green) or organic waste (brown). The yellow containers have a capacity of 3200 L and collect light and plastic packaging including bottles, cans, bricks, and aerosol sprays.

### Recycling plastic packages

Around 95.3% of the respondents in the pre-pilot group strongly agreed or agreed that transforming plastic packaging waste into new products is a valuable resource for the society, and 96.6% thought that reducing the use of plastics and increasing recycling preserves the environment and reduces landfill waste. A large share of the respondents (80.2%) believed that the recycling of plastics is a way to increase economic development (improve economic and social well-being) and 79.3% thought that increasing plastic recycling will create new jobs.

**Figure 3.**
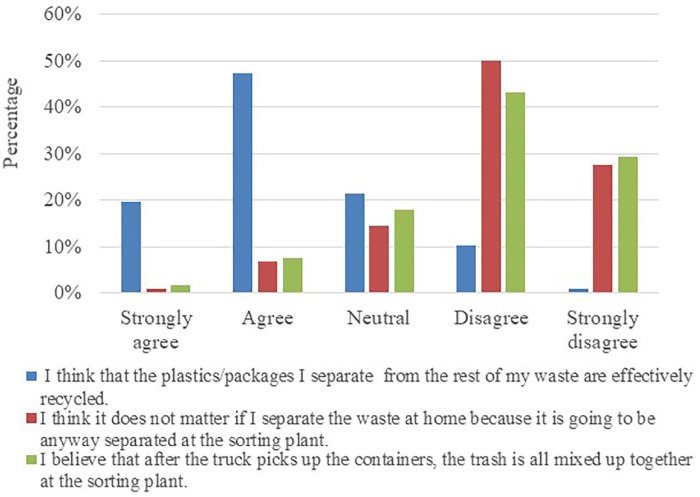
Pre-pilot group trust in actual handling of recyclables (*n* = 116).

Figure 3 shows their trust in the actual handling of recyclables by the municipality. About 67.2% thought that the packages they separate from the rest of their waste are effectively recycled (*n* = 78). Only 7.8% believed that the waste they separate at home is going to be separated again at the sorting plant (*n* = 9). About 9.5% thought that the trash is all mixed up together again at the sorting plant (*n* = 11) and 72.4% do not believe it. About 50.0% of the respondents in the pre-pilot group were aware of one or several plastic recycling initiatives in their municipality.

### Sorting and separating waste

Concerning the pre-pilot group experience with the domestic waste, 84.5% confirmed that they knew how to separate their waste according to the local law in their municipality. About 93.1% of the pre-pilot group stated that they always or often sort and separate the recyclable packages from the rest of their waste at home.

**Figure 4.**
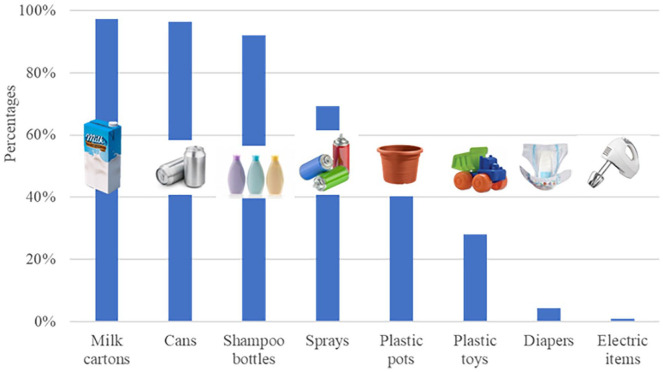
Items the pre-pilot group thought they could throw in the yellow container (*n* = 114).

The respondents were also asked to select one or several items they thought they could throw in the yellow container (*n* = 114). Figure 4 shows that the items that have been most selected by the respondents are milk cartons (97.4%), cans (96.5%), shampoo bottles (92.1%), sprays (69.3%), plastic pots (40.4%), and toys (28.1%). Items that the respondents thought they should not be put in the yellow container are diapers and electric items.

The packaging products were largely correctly identified by the respondents in the pre-pilot group. However, the introduction of products such as plastic pots, toys, diapers, and electric items in yellow containers would unfortunately increase the number of ‘rejected’ portions at the recycling station, reducing the effectiveness and efficiency of the whole recycling chain. It was therefore important to increase the knowledge of the participants during the PlastiCircle training sessions.

### Smart container

The smart container developed for the PlastiCircle project had several modules: an identification module, a label dispenser, a filling level sensor, vandalism protection features and a communication module. The identification module allowed use of NFC cards and mobile phones. An alphanumeric encryption system protects the information about the participants and complied with the European General Data Protection Regulation principles. Information including filling level of containers, user ID, internal temperature, number of labels available, and battery levels was sent by low-power wide-area network, to a gateway or receiver node installed in the neighbourhood.

**Figure 5.**
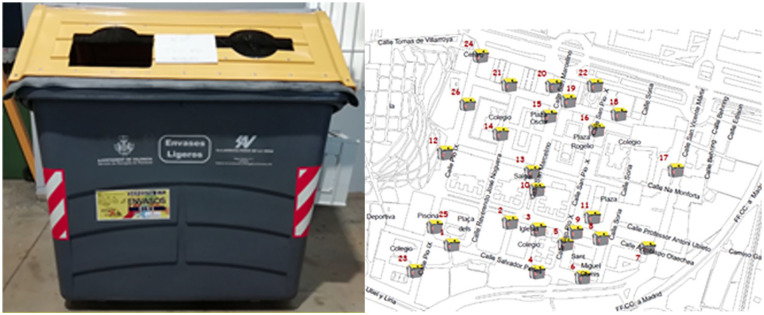
Locations of the containers in the San Marcelino district.

Figure 5 shows the location of the smart containers in the San Marcelino district.

Concerning the time it takes to go by foot from home to the closest container, 91.4% of the respondents of the post-pilot group stated that it takes between 2 and 5 min, and 6.9% between 5 and 10 min to walk to a container.

### Label dispenser

**Figure 6.**
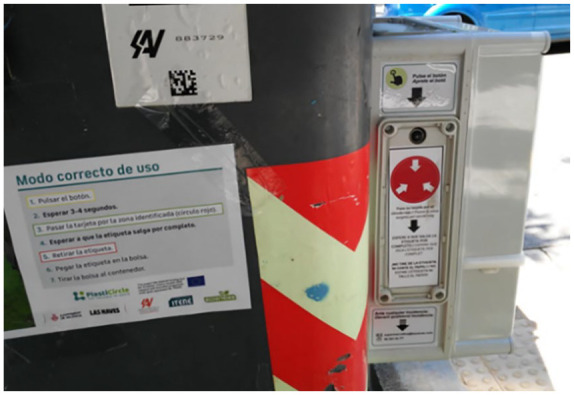
Yellow containers with label dispenser.

The label dispenser, developed by ITENE, fixed on the side of the containers read the card and printed a label (associated with the user ID). Figure 6 shows a label dispenser used in the project.

Of the 114 respondents in the post-pilot group, 71.9% stated that the label dispenser was very easy or somewhat easy to use (*n* = 82), whereas 16.7% thought the opposite (*n* = 19). The result demonstrates the efficiency of the label device.

The label dispenser was specially developed for the project and the Valencia pilot was the first to use this new technology. This is therefore not surprising that some adjustments were necessary at the beginning of the project. Table 2 presents some technical challenges experienced by the participants mostly at the beginning of the pilot. Most of the technical issues were due to malfunctions of the radio frequency identification card reading and damages caused by the rain and vandalism preventing the printing of labels by the dispenser. Occasionally, the participants also pulled out the label in the middle of the printing process, making the system inoperable for the next user.

**Table 2.** Respondents’ experience with the label dispenser (*n* = 19).

Experience with label device	Count	%
I had to go at least once to other containers nearby	16	84.2
I was unable to use once or several times the device because the label tape was broken, and the label was not coming out	15	78.9
I experienced once or several times that the label did not come out at all	14	73.7
I experienced that the label did not come out in one piece once or several times	9	47.4
I experienced once or several times that my ID keyring was not working properly	4	21.1
I think that it takes too much time for the label to come out from the device	4	21.1
It was difficult to keep the label stuck on the bag	1	5.3
I experienced once or several times that my user ID card was not working properly	1	5.3
Other comments	5	26.3

The technical problems were quickly identified at the beginning of the study and a 2.0 device version was developed by ITENE for the other pilot studies.

### Filling bags with recyclables

The bags were developed by INTERVAL S.A., a project partner involved in the recycling and transformation of the plastic. The total number of collected bags over the pilot weeks was 12,488: an average of 500 labelled bags was registered per week (71 bags per day). The results showed that 99.1% strongly agreed that it was easy to remember which recyclables they must put in the orange bags (*n* = 113). This is a good result for the pilot, showing the effectiveness and effectivity of the training sessions and communication actions organized by the Spanish partners of the project. In addition, 93.0% stated that the bags were easy to use (*n* = 106); 1.8% disagreed and commented that the bags could not be easily closed. One respondent specified that the bags were missing a cord to be easily closed.

Of the 114 respondents in the post-pilot group, 43.0% stated that it took them 3–4 days (*n* = 49), 28.9% 1–2 days (*n* = 33), and 19.3% 5–6 days (*n* = 22) to fill one bag. Only 8.8% stated that it took them one week or more (*n* = 10). The average number of persons living in households in the post-sample is 2.8 persons (SD = 1.0). A Kruskal–Wallis test shows that the number of days it took them to fill one bag decreases significantly as the number of persons living in their households increases (H=35.698, df=5, *p*<0.001).

The containers were collected three days a week (Tuesday, Thursday, and Saturday) according to the filling levels. The days after the collection of containers, Wednesdays (16.6%) and Fridays (16.2%) were the days with the highest numbers of labels printed and Saturday (11.7%) and Sunday (9.1%) the days with the smallest numbers. The respondents in the post-pilot group were asked how frequently they threw an orange bag in the yellow container. The results showed that 31.6% of the respondents threw a bag in the container once a week (*n* = 36), 30.7% once in three days (*n* = 35) and 25.4% once in two days (*n* = 29). A Kruskal–Wallis test shows that the frequency increases significantly with the number of persons living in their households (H=31.467 df=5, *p*<0.001).

### Content of the bags

SAV was responsible for examining the content of 741 labelled orange bags filled by 255 participants. The characterization included the evaluation of at least 35 labelled bags per week and 50 non-labelled bags per month. These bags were chosen randomly. Table 3 shows the materials to be collected in the yellow containers in the framework of the PlastiCircle project.

**Table 3.** Wanted and unwanted materials in the recycling containers.

Wanted materials	Unwanted materials
Bottles, beverage cans, tin cans, bricks, dairy packages, disposable plates and cups, cleaning products, plastic bags, plastic film, aluminium plates, trays, and foils, white trays and boxes, plastic caps left on the bottle, aerosol sprays, fruit boxes	Toys, home appliances, baby bottles, disposable cutlery, batteries, pens, lighters, disposable cutlery, rubber gloves, silicone moulds for pastries, clay pots or jars, natural cork stoppers, CD and DVD plastic cases, phone and tablet cases, mop buckets, medicine packets

The results showed that the percentage of unwanted materials in the labelled bags was 23.7% at the mid-term and 8.7% at the end of the pilot. The results confirmed a reduced behaviour-related uncertainty among the participants concerning the unwanted materials in the yellow containers. The results showed a higher rate of 27.5% of unwanted materials in the ordinary bags of non-participants. Table 4 shows the details of unwanted and wanted materials collected in the plastic packaging containers. The wanted materials included polyethylene terephthalate (PET) widely used for food packaging, high-density polyethylene (HDPE) used for plastic juice bottles, mixed or metal packages, bricks and plastic film.

**Table 4.** Percentages of wanted and unwanted materials in orange and ordinary bags.

	Participants	Non-participants
Wanted materials (%)		
PET packages	30.5	26.6
HDPE packages	8.0	7.8
Mixed packages	9.2	6.4
Metal packages	13.6	9.2
Bricks (food and beverages)	14.6	10.2
Film	15.5	12.3
Total wanted materials	91.3	72.5
Unwanted materials (%)		
Organic	1.5	2.4
Paper and cardboard	1.2	4.3
Wood	0.0	2.0
Glass	0.1	2.1
Metals	0.5	3.4
Textiles	1.0	5.6
Others	4.4	7.6
Total unwanted materials	8.7	27.5
Total materials	100.0	100.0

HDPE: high-density polyethylene; PET: polyethylene terephthalate.

Factors such as compaction, stacked materials and non-empty packages were considered in the compensation process: compaction as a positive factor because it reduces logistics costs; stacked packaging and non-empty packages as negative factors because stacking makes it impossible for the optical equipment to evaluate the type of waste materials and non-empty packages reduces the efficiency of the recycling process.

### Communication and workshops

Trained staff were present in the streets in the district two days a week in April to inform the citizens about the launch of the project with the aid of information stands. Communication actions before the pilot included one workshop at an elementary school with children and parents and two information stands at a medical centre for targeting women and older people and at a park for targeting families with children. Information was distributed, new participants were registered, and training sessions organized to explain how to sort the packages for the pilot. The communication plan also included several activities during the monitoring period, with information stands, four workshops for the residents of the San Marcelino district, talks with schoolchildren and the local press, television interviews, and an exhibition ‘Motius pel Canvi’ (Reasons for change) at the Rambleta Cultural Centre during July 2019 explaining the consequences of bad waste management on the environment. In addition, training sessions were organized at the neighbourhood association, every week from May to November (except in August), with the participants in order to teach them how to use the label device and the identification card.

Of the 114 respondents of the post-pilot group, 83.3% strongly agreed or agreed that the activities and workshops organized in the neighbourhood have been very useful to learn about which packages are recyclables and how to use the label device (*n* = 95). Around 15.8% had no opinion (*n* = 18).

### Compensation

The compensation system put in place in PlastiCircle included small prizes: bus tickets, sport event tickets, and products made with recycled materials (toys and bags). The prizes for the three participants having the largest numbers of points were three electric scooters. Previous studies also showed that to foster recycling, interventions should make the citizens identify themselves as recyclers, for example, by giving them positive feedback on their recycling rates (Nigbur et al., 2010). Of the 114 respondents of the post-pilot group, 96.5% strongly agreed or agreed that households should participate more in sorting recyclables. However, 87.1% of the pre-pilot group and 89.5% of the post-pilot group also stated that they should be rewarded for properly separating and throwing recyclables in the container. The results showed that rewarding the citizens (at least in the first stages of a new collection system) would be beneficial.

Table 5 shows which reward systems would be the most suitable for the participants. About 56.1% would like to get benefits for the whole neighbourhood, 50.9% to get discounts at local shops, 49.1% to pay less tax, 46.5% free transport tickets and 36.0% to get gifts. The ‘pant or pfand’ or consignment system for plastic bottles already in place in other countries (Norway, Sweden, Germany, and Denmark) was often mentioned in the comments left by the participants. The consumers received money back for the consigned bottles they returned to the shops.

**Table 5.** Post-pilot group opinion about the reward system.

Reward system	Count	%
Getting benefits for the whole neighbourhood	64	56.1
Discount coupons for local shops and services	58	50.9
Paying less tax	56	49.1
Public transport tickets	53	46.5
Getting points to exchange them against gifts	41	36.0
Other comments	4	3.5

### PAYT principle

In Spain, municipal solid waste taxes are not related to the consumption but to the particularities and characteristics of the households (Chamizo-González et al., 2016). There is therefore no difference regarding the quantity or quality of the waste they generate. Table 5 shows that 49.1% of the post-pilot group would like to be rewarded by paying less tax. There is no clear result concerning the respondents’ willingness to pay for the costs generated by the management of their waste; 50.0% of the post-sample strongly agreed or agreed that the citizens should pay according to the waste they generate, whereas 21.9% disagreed with the principle.

### General opinion on the PlastiCircle project

The results showed that 93.9% of the post-pilot group were satisfied with the project (*n* = 107), whereas 3.5% of the respondents had a negative opinion (*n* = 4).

Concerning what users like most about the project, the most repeated answers are related to the increase of consciousness in the neighbourhood. They positively agreed that the information provided on recyclables and good practices has been useful.

Regarding the negative aspects, the users highlighted the relatively short duration of the pilot; they would like this kind of initiative to be extended in order to have time to get used to the new sorting process at home. Some users positively commented on the positive local effect on families and the neighbourhood; however, they also did not perceive any impact at a city level. Other users commented that the PlastiCircle project focused too much on recycling and disregarded the whole value chain and the importance of reducing plastic consumption and reusing plastic at home.

## Discussion

The PlastiCircle project showed that household waste sorting and collection of recyclables could be improved in European cities and beyond. Greater efforts should be made to produce recyclables and to develop technologies to recycle more materials in new products. The current study contributes to understanding how to increase the recycling rates by encouraging the citizens to sort better their recyclables at home.

In demographic terms, women and older men with basic education levels are over-represented in our pilot groups, restricting the variability of the responses and limiting the correlations. Previous studies have shown that women are more likely to participate in return/recycling actions (Brough et al., 2016; Hall, 2014). Oztekin et al. (2017) also showed in a study among a Turkish university community that female attitudes were more innate (recycling is good, necessary, useful, and sensitive), whereas those of males were learnt (recycling is healthy, valuable, and correct). Our pilot groups may be constituted of environmentally sensitive participants who would have anyway participated in this project without reward.

The results obtained should be taken with caution in the case of extending the new recycling procedure to a city level. Communication interventions targeting other groups with less environmental concern and motivation should be developed to weaken negative recycling attitudes, uncertainty, or scepticism. For countries with early-stage recycling schemes, the focus should be on shaping the social norms for facilitating recycling behaviour (Cheung et al., 1999; Miliute-Plepiene et al., 2016).

The registration system was successful and the satisfactory number of 556 families was reached in PlastiCircle. An average of 200 users actively participated in actions and workshops. However, the six-month duration of the project and the summer holiday period had a discouraging effect. In addition, the gift exchange planned at the end of the pilot did not also retain their enthusiasm and motivation over the weeks. The visualization of accumulated points was not updated in real time, causing a certain scepticism and distrust in the compensation system. Real-time characterizations were not feasible because of the time gap between the container disposal to transport and further management.

A large share of the pre-pilot group was satisfied with the current domestic waste management system (68.1%) and the results confirmed a high trust of the citizens in the way the municipality handle the recyclables. However, they also need evidence that the products they put in the container are effectively recycled. Previous studies showed that consumers who believed that recycling contributes to benefits for the environment and society and were knowledgeable about potential consequences in recycling tend to have a positive attitude towards recycling (Cheung et al., 1999; Nigbur et al., 2010; Park and Ha, 2014; Seacat and Northrup, 2010). Positive attitudes provide the necessary driving force to constantly improve the collection of recyclables and increase the participation of the citizens. The participants were aware that more could be done concerning the sorting of recyclables at home and the use of recycled materials. They also need more information about the importance of the market demand for increasing the production of recyclables.

Information about the quality of raw materials that should be put in recycling containers is essential. The communication should be improved regarding the issues related to the recycling process and the reasons for the amount of plastic waste being incinerated and accumulated in landfills. Research showed that incentives and feedback were highly effective at reducing the contamination of recycling materials (Timlett and Williams, 2008; Vasileva and Ivanova, 2014). The compensation system put in place in the framework of PlastiCircle had the objective to motivate the participants to increase their knowledge and skills necessary to effectively recycle. Previous studies showed that the lack of opportunities, resources and skills may mitigate recycling behaviour (Mahmud and Osman, 2010; Tonglet et al., 2004).

A favourable attitude towards recycling, strong personal motivation and perceived ease of recycling are determinants of recycling intention (Ajzen, 1991). Tonglet et al. (2004) suggested that situational factors and making recycling easier may influence a positive attitude. The results of the present study showed that the citizens have a reasonable distance to walk to the closest container (under 5 min walk), which greatly facilitated the pilot study. The San Marcelino district is mostly constituted of apartments. The citizens put their trash bags in street containers when they know that the containers are empty and probably on their way to work or shops.

The participants were satisfied with the informative and training sessions they received during the pilot and the results showed less uncertainty and improved sorting waste to be put in the yellow containers. Previous studies showed that campaigns promoting recycling behaviour should include information and behavioural skills for promoting recycling (Seacat and Northrup, 2010).

Previous studies showed that perceived difficulty in recycling behaviour could moderate the intention to participate (Cheung et al., 1999). The labelled bag for the recyclables was found as useful and could be further improved by being biodegradable and with a cord to easily close it. Concerning the prototype of the label dispenser developed for the project, there were a few technical and vandalism issues at the beginning of the pilot. However, the whole system provided good results by facilitating the data collection for the compensation process.

Concerning data privacy, the participants did not have any major concerns. However, a system extended to the whole population may cause some resistance concerning the information of the individual household waste. This issue could be avoided by evaluating the waste at a neighbourhood level and by rewarding it with lower local taxes and infrastructure (plants, children’s play areas, etc.).

The municipalities often use a flat-rate system with no incentive to reduce the amount of waste produced by the population (Alzamora and Barros, 2020; Chamizo-González et al., 2016). The result indicated that for ensuring a successful long-term recycling rate, it will require to introduce monetary incentives to increase the participation in a new recycling program. This conclusion was also found in previous studies (Afroz et al., 2017; Siddique et al., 2010; Wagner, 2017).

The PAYT principle is based on the volume of waste that households generate and is difficult to apply (Botetzagias et al., 2020; Chamizo-González et al., 2018; Elia et al., 2015; Wagner, 2017). Recycling fees or consignment systems are also mentioned as solutions for increasing the level of returned recyclables (Yin et al., 2014). The participants of this study were willing to make efforts to reduce the amount of their non-recyclable waste if they can get some rewards and benefits. Public debates are recommended on charging solutions for polluters and non-recyclable waste reduction, adapted to the local reality and the household income levels.

## Conclusion

This paper conducts questionnaires during a pilot study in Valencia to explore the opinion and attitudes of citizens towards plastic packaging recycling. Citizens are essential in a circular economy approach: they can deliver dry and clean recyclables in the recycling containers and buy recycled products. In addition, they can also reduce their amount of waste.

The results of the present study showed that citizens have positive attitudes towards sorting recyclables at home to increase the number of recycled products. The impediment factors are the lack of citizens’ awareness and knowledge, as well as the lack of facilities for levering the recyclables.

The project provides municipalities with ideas for successful incentives and guidance for increasing awareness and participation in an effective recycling process. Communication between municipalities and citizens, as well as policy initiatives, are found to be facilitators for increasing the recycling rate.

The concept developed in PlastiCircle is based on training and rewarding the citizens for increasing the domestic collection of recyclables. The lack of awareness was handled by creating targeted campaigns and training sessions addressing the concerns of the citizens as well as educating them at an individual level. The education process about how to sort home waste and how improper waste affects the whole recycling chain has revealed to be useful by the citizens.

The pilot study in Valencia developed and tested smart technologies (filling level sensor, label dispenser and smart ID card) improving traditional domestic waste management practices. The association of smart technologies and targeted outreach campaigns provide municipalities with solutions for delivering efficient waste services, boosting the citizens’ participation, and promoting local economic development.
